# Veterinary Enhanced Recovery After Surgery (Vet-ERAS) Program in Dogs Undergoing Emergency Laparotomy

**DOI:** 10.3390/vetsci12040377

**Published:** 2025-04-17

**Authors:** Aida Fages Carcéles, Massimiliano Degani, Carme Soler, Claudio Iván Serra, Nuria Fernández-Salesa, Alejandra García de Carellán Mateo, Vicente José Herrería-Bustillo, Chiara Di Franco, Angela Briganti

**Affiliations:** 1 Department of Veterinary Sciences, Veterinary Teaching Hospital “M. Modenato”, University of Pisa, 56122 Pisa, Italy; 2 Doctoral School, Catholic University of Valencia “San Vicente Mártir”, 46018 Valencia, Spain; 3 Clinical Department of Companion Animal Sciences, Faculty of Veterinary Medicine, University of Liège, 4000 Liège, Belgium; 4 Small Animal Medicine and Surgery Department, Veterinary Teaching Hospital, Catholic University of Valencia “San Vicente Mártir”, 46018 Valencia, Spain

**Keywords:** critical care, surgery, perioperative complications, dogs

## Abstract

This study aimed to assess the efficacy of a veterinary enhanced recovery after surgery (Vet-ERAS) protocol in dogs undergoing emergency laparotomy (EL). The protocol, adapted from human medicine, encompassed a multidisciplinary approach and interventions such as early goal-directed fluid resuscitation, antibiotic prophylaxis, anti-emetic therapy, multimodal analgesia, and early enteral nutrition. A prospective observational study compared outcomes between dogs managed with the Vet-ERAS protocol (*n* = 59) and historical controls (*n* = 82). Implementation of the Vet-ERAS protocol resulted in a significant reduction in perioperative complication rates (*p* = 0.003) and 15-day mortality (5% vs. 20.7%). Intraoperative complications, including tachycardia and hypothermia, were significantly lower in the Vet-ERAS group. Compliance with the protocol improved over time (*p* = 0.01). These findings suggest that integrating the Vet-ERAS protocol into perioperative management can improve outcomes in dogs undergoing EL.

## Simple Summary

Our study aimed to evaluate the effectiveness of implementing a veterinary enhanced recovery after surgery (Vet-ERAS) protocol in dogs undergoing emergency laparotomy (EL). The Vet-ERAS protocol, adapted from successful strategies used in human medicine, was focused on optimizing perioperative care through evidence-based interventions. Compared to standard non-protocolized management previously adopted at the same institutions, dogs managed with the Vet-ERAS protocol experienced significantly lower perioperative complication rates and reduced mortality. Key components of the protocol included goal-directed fluid resuscitation, early administration of antibiotics, anti-emetic prophylaxis, multimodal analgesia, and early enteral nutrition. These findings suggest that integrating the Vet-ERAS protocol into the perioperative management of dogs undergoing EL could lead to improved outcomes and reduced morbidity, ultimately benefiting both veterinary patients and their owners.

## 1. Introduction

Enhanced recovery after surgery (ERAS^®^) is a perioperative care pathway for surgical human patients originally proposed for colonic resection [1] and promoted nowadays by the ERAS^®^ society [2]. It involves the application of evidence-based interventions throughout the preoperative, intraoperative, and postoperative periods, employing a multidisciplinary approach. ERAS^®^ programs aim to reduce perioperative metabolic stress by providing patient information and stabilization, optimizing pain management, and facilitating early enteral feeding and mobilization. The overarching goal is to enhance patient outcomes, minimize perioperative complications, and shorten hospitalization times [3].

Since its first application in patients undergoing colorectal surgery, ERAS^®^ programs have been widely implemented and recognized for their ability to reduce morbidity and mortality across various surgical procedures, including emergency laparotomy (EL) [4,5,6,7,8]. The term “EL” encompasses a wide group of surgeries, usually performed after a limited time for stabilization, to treat life-threating abdominal conditions, and it is widely recognized as a risk factor for anesthetic-related death and postoperative complications, both in human and veterinary medicine [9,10,11,12,13]. The 30-day mortality rate reported for humans ranges from 11% to 24% [14], with complications occurring 48% of the time compared with 27% for patients undergoing non-emergency surgery [15]. Challenges can arise in applying the ERAS^®^ approach to EL due to substantial differences in patient presentation, perioperative management, and disease severity [16]. Nonetheless, several studies have demonstrated promising outcomes following the implementation of ERAS^®^ protocols in the perioperative care of EL. Recent systematic reviews and meta-analyses in human medicine support the safe application of ERAS^®^ program in EL, resulting in reduced rates of postoperative complications, shorter hospital stays, and faster restoration of gastrointestinal function [8,17,18]. Guidelines for implementing ERAS^®^ protocols in EL patients have been published by corresponding study groups in recent years [19,20,21].

In veterinary medicine, EL is associated with high mortality and complication rates [11,12,13]. However, there is currently no published literature on the implementation of ERAS^®^ protocols in veterinary surgery. In a prior retrospective study involving 82 dogs undergoing EL at two different veterinary teaching hospitals, 15-day mortality and postoperative major complication rates were reported at 20.7% and 24.4%, respectively [12]. These findings provided sufficient data for the authors of the present study to develop a Vet-ERAS protocol aimed at optimizing recovery following EL surgery in this canine population. Therefore, this present study aimed to investigate the effectiveness and applicability of a Vet-ERAS program in dogs undergoing EL in the same veterinary teaching hospitals. We hypothesized that such a standardized multidisciplinary perioperative protocol, adapted from the ERAS^®^ programs used in human medicine, would (1) reduce perioperative complications and (2) reduce mortality rates following EL in our canine population compared to standard non-protocolized management previously adopted at the same institutions.

## 2. Materials and Methods

### 2.1. Study Design

Following the completion of a retrospective study [12], we conducted an observational prospective trial at the veterinary teaching hospitals of the University of Pisa and the Catholic University of Valencia, involving all dogs undergoing EL (EL). The study received approval from the Ethics Committee of the University of Pisa (Nr. 12/2021).

### 2.2. Vet-ERAS Protocol Development

A multidisciplinary team comprising surgeons, anesthesiologists, and critical care specialists from both institutions collaborated to develop a Vet-ERAS protocol tailored for dogs undergoing EL. Drawing inspiration from protocols applied in human medicine, key elements were extrapolated, including the use of early warning scores for immediate goal-directed fluid resuscitation, early identification of sepsis with prompt administration of antibiotics, and the involvement of various specialists in postoperative care to ensure optimal pain management, early nutrition, and mobilization. Additional considerations for protocol development included insights from existing veterinary literature on perioperative management in dogs undergoing abdominal surgery, the expertise of the multidisciplinary team, and data obtained from the previous retrospective study [12]. The final Vet- ERAS^®^ program for dogs undergoing EL (Table S1) incorporated a perioperative surgical safety checklist (Table S2) to ensure adherence to protocol guidelines. Table 1 highlights the key components of the protocol.

An early goal-directed fluid resuscitation was initiated for stabilization in case of hypovolemic shock upon arrival [22]. Dogs meeting the preoperative acceptable resuscitation endpoints were deemed stable from a cardiovascular point of view and transferred to surgery (Table 2), except for cases in which delaying the procedure would pose a greater risk to the stability of the dog, such as sepsis or active hemorrhage.

Fluids were administered according to protocol recommendations. Isotonic crystalloids were the first-line fluid choice. Hypertonic saline was used selectively in hypovolemic patients when rapid volume expansion was required or total fluid volume needed to be minimized. The choice and titration were at the discretion of the attending criticalist.

Dogs in shock also received oxygen supplementation via a mask to improve tissue oxygen saturation [23]. The protocol mandated the use of blood lactate concentration measurements as a marker of tissue perfusion and resuscitation response [24,25,26]. In cases of hematocrit <20% or hemoglobin concentration <6 g/dL, a blood transfusion was administered. All dogs received antibiotic prophylaxis 30 min before surgery, following the respective institution’s guidelines. Anti-emetic prophylaxis, comprising maropitant at 1 mg/kg administered intravenously (IV) or subcutaneously, was also included in the protocol, given that EL has been identified as a risk factor for postoperative regurgitation and vomiting in dogs [27]. Detailed information was provided to the owners through discussions involving the anesthetist, criticalist, and surgeon responsible for the case. Owners were informed about the prognosis, costs, risks, and potential complications associated with the procedure, and written consent was obtained for all dogs. The anesthetic protocol was titrated according to the individual patient’s condition by the anesthetist in charge of the case.

Perioperative pain management was based on a multimodal opioid-sparing approach. One of the locoregional anesthesia techniques recommended by the Vet-ERAS protocol (Table 3) had to be performed in all patients, either before or after surgery. The choice of technique was determined by the attending anesthetist. Systemic multimodal analgesia was individualized based on patient status and clinician preference, and included constant rate infusion of ketamine, dexmedetomidine, opioids (methadone, fentanyl), and lidocaine, the latter administered intraoperatively when the chosen locoregional technique was performed at the end of surgery. Non-steroidal anti-inflammatory drugs (NSAIDs) were also used, but only in the absence of contraindications. All surgeries were performed by experienced surgical teams, including board-certified surgeons or residents under supervision, following standardized surgical techniques.

Postoperative management included blood lactate concentration measurements to assess tissue perfusion during recovery and guide fluid administration. Postoperative pain was assessed using the short form of the Glasgow Composite Pain Scale (SF-GCPS) [28]. The attending anesthetist and criticalist established an individualized postoperative analgesic plan and provided instructions regarding rescue analgesia administration. Enteral nutrition was administered to all animals according to the scheme outlined in Table 4. The nutritional plan was tailored to each dog based on their resting energy requirements (RER), which could be calculated using linear or exponential formulas [29]. Prior to the conclusion of anesthesia, an esophagostomy feeding tube was placed in all dogs deemed critically ill or with a history of anorexia. In case an esophagostomy feeding tube was not preemptively placed, a nasogastric tube was inserted under sedation in dogs presenting with anorexia during the postoperative period. Finally, the protocol recommended early mobilization of the dog, scheduling the first walk 6 h post-surgery whenever feasible. Implementation of this protocol in the perioperative management of dogs undergoing EL in both hospitals was overseen by members of the multidisciplinary team, with regular meetings conducted with staff involved in patient management at the respective institutions.

### 2.3. Vet-ERAS Protocol Implementation

The Vet-ERAS protocol was implemented in the perioperative management of all dogs undergoing emergency laparotomy (EL) at the two participating veterinary teaching hospitals over a 12-month period. These dogs were prospectively enrolled and constituted the Vet-ERAS group. EL was defined as any unplanned surgical exploration of the abdomen, due to different underlying conditions such as obstructive foreign body/neoplasia, pyometra, gastric-dilation volvulus (GDV), and hemo- or uroperitoneum. Dogs undergoing cesarean sections were not included in this study. Each dog underwent clinical examination on arrival, including body condition score (BCS) on a five-point scale evaluation (measured by a 5-point scale 1 to 5, 1: emaciated, 2: lean, 3: ideal, 4: overweight, and 5: obese). Physical examination [heart rate (HR), respiratory rate (RR), systolic arterial pressure (SAP), and rectal body temperature (T°)] and perioperative laboratory findings (hematology, biochemistry, and blood gas analysis) were recorded. Shock index (SI) (HR/SAP), American Society of Anesthesiologists (ASA) physical status, and modified Charlson comorbidity index (CCI) [12] were calculated. Systemic inflammatory response syndrome (SIRS), sepsis, acute kidney injury, and respiratory failure were diagnosed based on predefined criteria [12]. Major electrolyte imbalances were defined as K^+^ < 3.3 or >5.2 mmol/L, Ca^2^^+^ < 1.1 or >1.6 mmol/L, Na^+^ < 125 or >165 mmol/L, Cl^−^ < 90 or >140 mmol/L, in accordance with the thresholds used in our previous retrospective study [12].

Anti-emetic prophylaxis, duration of surgery and anesthesia, administration of blood product transfusion, use of locoregional anesthesia techniques, and time to first oral intake were recorded. Perioperative complications were classified using the same system as in the previous retrospective study [12]. Intraoperative complications considered were hypotension [mean arterial blood pressure (MAP) < 60 mmHg], tachycardia [HR > 130 beats per minute (bpm)], bradycardia (HR < 50 bpm), and hypothermia (rectal T° < 36 °C). The same classification for postoperative complications as utilized by Fages et al. [12] was used in this prospective study. Fifteen-day mortality and length of hospitalization time were both recorded. Additionally, compliance with the Vet-ERAS protocol was retrospectively evaluated. Compliance with the protocol was assessed based on the number of key components of the Vet-ERAS protocol (Table 1) not accomplished, categorized as good (0–2), moderate (3–4), or bad (>4). For the purpose of compliance analysis, the study period was divided into the following two phases: phase 1 corresponding to the first 6 months of implementation (T_6_) and phase 2 corresponding to the final 6 months of the study (T_end_).

### 2.4. Comparison with Pre-Protocol Implementation Outcomes

The results of this trial were compared with those of the previous retrospective study, which included 82 dogs undergoing EL at the two veterinary teaching hospitals from January 2018 to March 2020 (group CONTROL) [12]. Fifteen-day mortality, perioperative complications rate, and length of hospitalization time were compared between group Vet-ERAS and group CONTROL. Conditions requiring EL, physical examination parameters on admission, preoperative laboratory findings, use of antiemetic prophylaxis, duration of surgery and anesthesia, administration of blood product transfusion, use of locoregional anesthesia techniques, and time to first oral intake were compared between the groups as well.

### 2.5. Statistical Analysis

The sample size was calculated based on the reduction in the complications and the mortality rate. With α of 0.05, β error of 0.2, and assuming a 70% reduction in the complications and a 65% decrease in mortality in the Vet-ERAS group, a minimum of 57 dogs were required to detect a statistically significant difference. To account for potential data loss, the minimum number was increased to 60 dogs. Descriptive statistics were calculated and expressed as percentages. Data distribution was analyzed using the D’Agostino–Pearson test. Parametrically normally distributed data were expressed as mean and standard deviation (SD), while non-parametric and non-normally distributed variables were expressed as median (range). Either Student’s *t*-test or the Mann–Whitney test was used for between-group comparisons. Statistical significance was set at *p* < 0.05. The Chi-square test was employed to compare variable incidences between groups, with results reported as odds ratios (ORs). A multiple logistic regression was used to evaluate the association of all of the perioperative and intraoperative variables with mortality and postoperative complications. Results are reported as odds ratio (OR). Prism Version 10.0 (GraphPad Software Inc., San Diego, CA, USA) were used for data analysis.

## 3. Results

Of the 60 dogs enrolled, 59 were included in group Vet-ERAS. One case was excluded from analysis due to incomplete data recording. Demographic data, including age, sex, weight, body condition score (BCS), and underlying conditions necessitating EL, are summarized in Table 5. There were no significant differences between the two groups regarding demographic characteristics. In the Vet-ERAS group, a significantly higher number of dogs underwent EL due to obstructive gastrointestinal neoplasia and hemoperitoneum, while pyometra and uroperitoneum were significantly more frequent in the CONTROL group (*p* < 0.05). Septic peritonitis was diagnosed in 10 out of 59 dogs (16.9%) in the Vet-ERAS group and in 11 out of 82 dogs (13.4%) in the CONTROL group, without a significant difference. There was no significant difference in the duration of anesthesia and surgery between the groups (see Table 5).

The mortality rate in the Vet-ERAS group was 5%, which represented a significant reduction (*p* = 0.01) compared to the CONTROL group, where the 15-day mortality rate was 20.7% [12]. Among the Vet-ERAS group, 3 out of 59 dogs died during the perioperative period: 1 during surgery, 2 within 24 to 72 h post-surgery, the remainder died between 72 and 198 h. No dog was euthanized or died after discharge within the 15-day postoperative period. Table 6 presents data on perioperative complications in both groups. Significantly fewer dogs in the Vet-ERAS group experienced perioperative complications compared to the CONTROL group (*p* = 0.003). Specifically, intraoperative complications such as tachycardia and hypothermia were significantly less frequent in the Vet-ERAS group. In the postoperative period, the incidence of minor complications was significantly lower in the Vet-ERAS group compared to the CONTROL group (45.8% versus 80.5%, *p* = 0.003). However, there were no statistically significant differences in hospitalization time between the two groups (median 3 days in both groups). The multiple logistic analysis found the intraoperative hypotension was the only factor associated with major complications, with an odds ratio of 3.03 (95% CI: 1.0116–16.48), while the absence of hypoalbuminemia was significantly associated with a reduced risk of complications (OR = 0.058; 95% CI: 0.004–0.439).

Preoperative physical examination parameters, laboratory findings, and the ASA physical status did not differ significantly between the Vet-ERAS and CONTROL groups. Notably, a higher proportion of dogs in the Vet-ERAS group had no comorbidities compared to the CONTROL group (*p* < 0.001). Data regarding preoperative physical examination parameters and laboratory findings, ASA status and CCI, lactate blood concentration, and SI are reported in Table 7. The goal-directed fluid resuscitation strategy used in the Vet-ERAS group produced a significant reduction (*p* < 0.0001) in lactate blood concentration [1.4 (0.3–8.1) versus 2.5 (0.6–12.8)] and SI [0.7 (0.33–1.2) versus 0.99 (0.33–3.5)] in comparison with respective pre-stabilization values. Among the 59 dogs included in the Vet-ERAS group, 17 (28.8%) underwent surgery despite not fully meeting the predefined stabilization endpoints. Specifically, four dogs had persistently increased lactate concentrations after fluid therapy resuscitation, eight showed decreasing lactate levels that remained above the target threshold, three remained hypothermic, and two were hyperthermic at the time of surgery. Blood transfusions were administered to 15/59 dogs (25.4%) in the Vet-ERAS group and in 18/82 dogs (21.9%) in the CONTROL group, without statistically significant differences. As expected, the use of anti-emetic prophylaxis, locoregional anesthesia techniques, and feeding tubes was significantly more frequent in the Vet-ERAS group (*p* < 0.0001), reflecting the protocol’s structured implementation and adherence. Data are reported in Table 8. A total of 29 dogs were included in phase 1 and 30 dogs were included in phase 2. The Vet-ERAS protocol compliance at T_6_ was good in 13/29 cases (44.8%), moderate in 14/29 cases (48.3%), and bad in 2/29 cases (6.9%). Compliance at T_end_ was good in 24/30 cases (80%), moderate in 5/30 cases (16.7%), and bad in 1/30 cases (3.3%), showing a significant improvement between phase 1 and phase 2 (*p* = 0.01).

## 4. Discussion

Based on the findings of this study, the introduction of a Vet-ERAS protocol in the management of dogs undergoing EL at two distinct veterinary teaching hospitals led to decreased 15-day mortality rates and minor postoperative complication rates compared to dogs treated using a non-protocolized care pathway. This reduction not only highlights the potential life-saving impact of the ERAS^®^ approach in veterinary medicine but also underscores the importance of a multidisciplinary, evidence-based approach to perioperative care.

Emergency major abdominal surgery is extensively studied in human literature due to its high risk of morbidity and mortality [15,30,31,32]. In a previous retrospective study, we reported a mortality rate of 20.7% and an overall postoperative complication rate of 80.5% in dogs undergoing EL. To our knowledge, no comparable studies exist in veterinary literature regarding the implementation of perioperative quality improvement programs. While some studies have documented benefits of surgical safety checklists in decreasing perioperative complications, their impact on mortality remains undocumented [33,34]. In our study, mortality decreased from 20.7% to 5%, although various factors could have influenced this outcome. The Vet-ERAS group exhibited a higher number of patients with lower number of comorbidities, and there were significant differences in the types of surgeries performed between groups. This imbalance may have contributed, at least in part, to the observed reduction in mortality and might represent a relevant confounding factor. While the prospective nature of the Vet-ERAS group and the retrospective design of the CONTROL group limit direct comparability, future randomized studies are warranted to further clarify this relationship.

Identifying specific reasons for the reduction in mortality requires further investigation with larger sample sizes to elucidate how comorbidities may influence survival. The lower incidence of perioperative complications observed in the Vet-ERAS group underscores the effectiveness of the protocol in mitigating postoperative morbidity. In our study, we observed a significant reduction in postoperative minor complications in the group following the Vet-ERAS protocol compared to the CONTROL group. The protocol likely emphasized measures aimed at preventing minor complications, such as early mobilization, optimized pain management, and enhanced nutrition. These interventions may have been effective in reducing minor complications such as wound infections, urinary retention, or minor gastrointestinal disturbances. However, there was not a significant difference in the occurrence of major complications between the two groups. Major complications such as SIRS, acute kidney injury or respiratory failure, may be influenced by factors beyond the scope of this Vet-ERAS protocol, such as the complexity of the surgical procedure, underlying comorbidities, or unforeseen intraoperative events. These findings suggest that the protocol was more effective in improving perioperative parameters that are modifiable, such as thermoregulation and hemodynamic stability, than in preventing complications primarily related to the underlying disease severity. As highlighted by the multiple logistic regression analysis, intraoperative hypotension was a factor associated with major complications. It is possible that the intraoperative hemodynamic stabilization was not optimized during the study, and in fact the protocol was mainly focused on implementing the preoperative stabilization. When comparing the incidence of intraoperative hypotension of the two groups, no reduction was obtained by the Vet-ERAS protocol, unlike the incidence of tachycardia and hypothermia that was significantly lower in the Vet-ERAS group. This result highlights the need to continue to improve the Vet-ERAS protocol to manage intraoperative hemodynamic stabilization of these patients. While our Vet-ERAS protocol may have optimized certain aspects of perioperative care, it may not have directly addressed factors contributing to major complications. Additionally, the sample size of our study may have limited our ability to detect differences in major complication rates between the two groups. Further research is needed to elucidate the specific mechanisms underlying the observed reduction in minor complications and the lack of significant impact on major complications in the Vet-ERAS group. This could involve detailed analysis of individual complication types, exploration of patient-specific factors, and consideration of additional perioperative interventions that may further mitigate major complication risks.

Notably, the significant decrease in intraoperative complications such as tachycardia and hypothermia suggest that the Vet-ERAS approach may contribute to improved intraoperative stability and patient safety. This is particularly relevant in the context of EL, where rapid and effective perioperative management is essential for patient survival. Prior to surgery, significant reductions were observed in SI and lactate values within the Vet-ERAS group. Ensuring preoperative patient stabilization was a primary objective of the protocol, potentially aiding in the mitigation of tachycardia during surgery. One of the cornerstones of our Vet-ERAS protocol is the emphasis on goal-directed fluid resuscitation strategies to optimize hemodynamic stability and tissue perfusion. Our findings demonstrate significant improvements in markers of tissue perfusion, including reductions in lactate blood concentration and shock index, following the implementation of goal-directed fluid resuscitation. This suggests that tailored fluid management strategies, guided by physiological parameters, play a crucial role in optimizing patient outcomes in the perioperative period. Maintaining normothermia aligns with ERAS^®^ protocols in human medicine [3], supported by evidence demonstrating its efficacy in reducing wound infections, cardiac complications, and bleeding [35,36,37]. In our study, the use of forced-air heating covers was recommended by the checklist and possibly contributed to the decrease in intraoperative hypothermia among dogs in the Vet-ERAS group. The occurrence of intraoperative hypotension did not show a reduction post-protocol implementation. Variability in anesthetic protocols may have influenced the incidence of intraoperative complications, particularly hypotension. However, this reflects the real-world nature of emergency case management in clinical settings, where anesthetic plans are necessarily individualized and adjusted in real time. Nonetheless, due to insufficient data in the retrospective study, the duration of intraoperative hypotension and the response to treatment were not assessed. Intraoperative hypotension has been linked to mortality in our retrospective study [12] and is documented to increase perioperative morbidity and mortality in both veterinary and human studies [38,39,40,41]. Following protocol implementation, 64.4% of patients experienced intraoperative hypotension; however, mortality and morbidity rates were significantly decreased compared to the CONTROL group. Given the limited number of cases in the Vet-ERAS group, further investigations are warranted to fully evaluate the true impact and identify strategies for mitigating this significant complication.

Multimodal pain management, with a focus on minimizing opioid use to reduce systemic adverse effects and the risk of postoperative ileus development, constitutes a pivotal aspect of ERAS protocols in human medicine [42,43,44]. Multimodal pain management and effective analgesia provision are recognized as essential components of optimal patient care in veterinary medicine [45,46]. Locoregional anesthesia has been shown to effectively prevent both intraoperative nociception and postoperative pain while reducing potential adverse effects associated with systemic opioid administration [47,48,49,50]. In a recent worldwide multicenter study published by Redondo et al. [13], locoregional anesthesia was found to be associated with decreased mortality in dogs. In a recent worldwide multicenter study published by Redondo et al. [13], the use of locoregional techniques was associated with decreased mortality in dogs; however, this finding may be partially influenced by confounding factors such as the level of expertise of the anesthetist, as more experienced clinicians are more likely to perform these techniques. The present study has shown the safety of locoregional anesthesia in dogs undergoing emergency abdominal surgery. Given that this population is often presented in shock conditions, the use of locoregional anesthesia techniques can reduce the need for systemic analgesics and contribute to a more hemodynamically stable perioperative course. Their incorporation should be considered an integral part of perioperative quality improvement protocols.

Aggressive treatment of postoperative nausea and vomiting (PONV) constitutes another crucial component of ERAS protocols in human medicine [3]. PONV is one of the most frequently reported postoperative complaints and may hinder recovery [2]. Multimodal strategies to mitigate these complications include opioid-sparing techniques (such as locoregional anesthesia and NSAIDs) and prophylactic treatment with antiemetic drugs [2]. In dogs, emergency surgery has been identified as a risk factor for postoperative regurgitation and vomiting [27]. Consequently, our protocol included prophylactic treatment with maropitant, alongside other strategies such as multimodal pain management to reduce opioid drug use. However, postoperative vomiting was not significantly reduced in the Vet-ERAS group compared to the CONTROL group. In veterinary patients, it is challenging to ascertain when an animal is experiencing nausea, and subtle symptoms like ptyalism were not recorded in this study. It is possible that, even without a significant reduction in the percentage of dogs experiencing vomiting, the protocol indirectly facilitated earlier initiation of oral intake. Further studies are warranted to fully evaluate the necessity of maropitant prophylaxis treatment in this population.

We observed a significant reduction in the time from surgery to first oral intake with the implementation of the Vet-ERAS protocol. Our program mandated a nutritional plan for all animals postoperatively, with feeding tubes placed in critical patients. While we cannot establish direct causal relationships, these factors likely played a crucial role in facilitating earlier initiation of oral intake in the Vet-ERAS group. Early nutritional support has been demonstrated to enhance outcomes and expedite recovery in dogs [51] and constitutes a primary objective of ERAS protocols in human medicine [3,4,5,6,7,8,9,10,11,12,13,14,15,16,17,18,19,20,21,22,23,24,25,26,27,28,29,30,31,32,33,34,35,36,37,38,39,40,41,42,43,44,45,46,47,48,49,50,51,52]. Early enteral nutrition is advocated to commence as soon as possible, offering numerous benefits such as modulating the inflammatory response and metabolism, preserving gut integrity, and stimulating bowel movements, thereby reducing postoperative ileus [53,54]. Although specific conclusions regarding our results on early oral intake and improved final outcomes cannot be drawn, considering the aforementioned factors, early oral intake should be a focal point and our protocol could be instrumental in enhancing outcomes in these patients.

Interestingly, a significantly higher proportion of patients experienced hyperglycemia in the postoperative period in the CONTROL group compared to the Vet-ERAS group (62% vs. 39.6%). While poorly reported in veterinary literature, surgery-induced insulin resistance has been extensively studied in human medicine [55,56]. Vet-ERAS interventions primarily aim to mitigate surgical stress and modulate perioperative insulin resistance [52]. The “stress response” encompasses hormonal and metabolic changes occurring after injury or surgery, often exacerbated by postoperative complications [56]. Although hormonal effects vary and remain incompletely understood, insulin resistance, characterized by reduced insulin effectiveness affecting glucose, lipid, and protein metabolism, constitutes a key mechanism underlying common surgical complications [52,55,56]. While specific conclusions regarding our findings cannot be drawn, our interventions may have led to an overall decrease in surgical stress response and indirectly improved hormonal homeostasis regarding insulin resistance, resulting in fewer patients developing postoperative hyperglycemia and contributing to the overall reduction in minor postoperative complications. The observed reduction in hyperglycemic episodes in the Vet-ERAS group may be partially explained by improved modulation of the surgical stress response through multimodal analgesia. In particular, the use of locoregional techniques has been shown to suppress glycemic and cortisol elevations post-surgery, likely by blocking afferent nociceptive input at the peripheral level and reducing activation of the hypothalamic–pituitary–adrenal axis [57].

Although the Vet-ERAS protocol significantly reduced both postoperative complications and short-term mortality, no difference was observed in the length of hospitalization between the two groups. Several factors may explain this finding. Discharge decisions in veterinary medicine may be influenced by non-medical factors, such as owner availability or logistical constraints, which may limit the ability to expedite discharge even in patients recovering well. In addition, postoperative monitoring is often prolonged after EL, regardless of complication severity, due to the critical nature of the underlying conditions and the need to ensure stability before discharge.

It is noteworthy that the compliance with the Vet-ERAS protocol improved over the course of the study, suggesting that ongoing education and support are crucial for successful protocol implementation. The overall compliance of the protocol at the end of the study period was rated as good in 62.7% of cases, moderate in 32.7%, and poor in 1.7%. Despite efforts, several elements of the protocol were not consistently performed in all patients. Achieving optimal compliance with a protocol, particularly during its early implementation phase, poses challenges. Implementing changes in treatment pathways, such as ERAS protocols, often requires time and faces obstacles to full adherence [58]. Encouragingly, in our study, better adherence to the protocol was observed during the second implementation phase, underscoring the notion that improvement efforts necessitate time to solidify [59,60]. Regular audits serve as vital learning opportunities to understand the challenges encountered in implementing quality improvement programs and are integral to every ERAS initiative [3,59]. Utilizing frequent and timely feedback has been shown to be instrumental in successful ERAS implementation efforts [60]. Multiple audits involving all staff managing these patients were conducted in our project. Continuous staff education, along with discussions on outcomes, endpoints, and protocol adherence, are essential to enhance protocol adherence [59].

This study has several limitations that should be acknowledged. Firstly, the number of patients was calculated to individuate a considerable reduction in complications and mortality, 70% and 65%, respectively. Further studies with a higher number of patients would be able to also reveal smaller improvements in patients’ outcomes. Secondly, our study was conducted at two veterinary teaching hospitals, which may limit the generalizability of our findings to other clinical settings. Variability in patient populations, surgical techniques, and perioperative practices across different institutions could potentially influence the applicability of the Vet-ERAS protocol in various veterinary contexts. Additionally, the retrospective nature of our study design for the CONTROL group may introduce inherent biases and confounding variables that could impact the comparability of the two study groups. A mild degree of selection bias in the Vet-ERAS group cannot be completely excluded. Although the protocol allowed exceptions to preoperative resuscitation targets at the discretion of the attending criticalist, dogs in the Vet-ERAS group were overall more stable at the time of surgery. This likely reflects the effectiveness of structured stabilization and improved preoperative assessment, including shared decision-making with owners, rather than deliberate exclusion of unstable patients. Enhanced owner communication may also have contributed to more appropriate case selection, possibly leading to the avoidance of futile surgery in patients with very poor prognosis. While this introduces a form of selection bias, it also represents a positive outcome of structured, ethical perioperative care that emphasizes shared decision-making. Although stabilization goals and inclusion criteria were clearly defined, the variability inherent to clinical decision-making may have influenced case inclusion and perioperative management. Furthermore, while we observed a significant reduction in perioperative complications and mortality rates in the Vet-ERAS group, it is important to recognize that other unmeasured variables or external factors may have influenced these outcomes. Factors such as surgeon experience, patient comorbidities, and individual variations in perioperative care could potentially confound the observed associations between the Vet-RAS protocol and patient outcomes. Another limitation is the relatively small sample size of our study, which may limit the statistical power to detect smaller differences in outcomes between the Vet-ERAS and CONTROL groups. Despite calculating the sample size based on anticipated reductions in mortality, larger studies with a more extensive patient cohort would be beneficial to further validate our findings and provide more robust evidence. Additionally, the assessment of compliance with the Vet-ERAS protocol relied on retrospective data collection, which may introduce inaccuracies and recall bias. Another limit was the 15 days postoperative mortality evaluation; in human medicine it has been demonstrated that a severe perioperative complication can impact the long-term survival of the patients [61,62]. Future studies incorporating a higher number of patients and hospitals, longer postoperative mortality or survival, and standardized compliance assessment tools could provide more reliable insights into the implementation of the Vet-ERAS protocol.

## 5. Conclusions

In conclusion, the present study demonstrates that structured, multidisciplinary perioperative care—including systematic preoperative stabilization—can significantly improve outcomes in dogs undergoing emergency laparotomy. The Vet-ERAS protocol, as a standardized approach inspired by the ERAS^®^ principles in human medicine, contributed to a reduction in postoperative complications and short-term mortality. These findings support the integration of coordinated, evidence-based perioperative strategies in veterinary surgical practice. The Vet-ERAS protocol does not introduce novel clinical techniques but rather reinforces and standardizes existing best practices in a structured format. Its value may be most evident in clinical settings where perioperative care is less uniform, or where team experience varies. Further studies are needed to validate and expand the use of ERAS^®^-inspired protocols in veterinary medicine.

## Figures and Tables

**Table 1:** Key components of the veterinary enhanced recovery after surgery (Vet-ERAS) protocol in dogs undergoing emergency laparotomy, during the different perioperative phases.

Period	Vet-ERAS Protocol Key Components
Perioperative	Use of the surgical safety checklist
Preoperative	▪ Stabilization through endpoints ▪ Blood lactate concentration measurements ▪ Antiemetic prophylaxis ▪ Antibiotic prophylaxis ▪ Owner information and education
Intraoperative	▪ Locoregional anesthesia ▪ Communication anesthetist—surgeon ▪ Sponge count ▪ Feeding tube placement before recovery in critically ill patients
Postoperative	▪ Analgesic plan prepared by anesthetist and criticalist ▪ Lactate measurements ▪ Early nutrition mobilization

**Table 2:** Vet-ERAS protocol acceptable resuscitation endpoints, adapted from Hammond et al. [22]. HR, heart rate (beats per minute); RR, respiratory rate (breaths per minute); SAP (systolic blood pressure); MAP, mean arterial pressure; T° (rectal body temperature).

Resuscitation Endpoints	
HR (bpm)	Small-breed dogs: 100–160 Large-breed dogs: 60–100
RR (bpm)	20–40
SAP (mmHg)	>100
MAP (mmHg)	>70
Shock index	<1
T (°C)	37.5–39.1
Lactate blood concentration (mmol/L)	<2.5

**Table 3:** Locoregional anesthesia techniques and corresponding drugs included in the Vet-ERAS protocol used in this study. QL, quadratus lumborum; TAP, transversus abdominis plane; RSP, rectus sheath plane.

Locoregional Anesthesia Techniques	Drugs
Epidural injection	Ropivacaine or bupivacaine 0.5% + morphine 0.1 mg/kg Morphine 0.1 mg/kg
Inter-fascial plane blocks (QL, TAP, RSP block)	Ropivacaine or bupivacaine (3 mg/kg)
Linea alba infiltration	Ropivacaine or bupivacaine 0.5% (3 mg/kg)
Intraperitoneal lavage	Ropivacaine or bupivacaine 0.5% (3 mg/kg)

**Table 4:** Nutritional plan, adapted from Corbee and Kerkhoven [29].

NUTRITIONAL PLAN
▪ First meal to be offered 6–12 h after surgery. ▪ Voluntary nutrition to be preferred, otherwise feeding tube, if placed. ▪ Three meals per day with the RER gradually increased over 2–4 days: 33% RER, 66% RER, 100% RER.

**Table 5:** Age, sex, weight, BCS, and the different conditions requiring EL of dogs in group Vet-ERAS and group CONTROL. Data regarding age, weight, and the duration of anesthesia and surgery are shown as median and range. * Significant difference between the two groups (*p* < 0.05).

	Vet-ERAS (n = 59)	CONTROL (n = 82)	*p* Value
Age (years)	8 (0.5–17)	7.1 (04–15.6)	0.62
Sex:			
Male (n, %)	33 (56)	37 (45)	0.2
Female (n, %)	26 (44)	45 (55)	0.2
Weight (kg)	22 (2.45–60)	18 (1.7–50)	0.27
BCS (1–5 scale):			
≤2 (n, %)	10 (16.9)	17 (20.7)	0.76
3 (n, %)	39 (66.1)	54 (65.8)	0.76
≥4 (n, %)	10 (16.9)	11 (13.4)	0.76
Conditions requiring EL (n, %):			
▪ Gastrointestinal for obstructive foreign body	22 (37.3)	34 (41.5)	0.45
▪ Gastrointestinal for obstructive tumoral mass	4 (6.8)	0	<0.05 *
▪ Pyometra	5 (8.5)	15 (18.3)	<0.05 *
▪ Gastric dilatation-volvulus (GDV)	13 (22)	15 (18.3)	0.42
▪ Hemoperitoneum	8 (13.6)	2 (2.4)	<0.05 *
▪ Uroperitoneum	1 (1.7)	8 (9.7)	<0.05 *
▪ Exploratory laparotomy (other)	6 (10.6)	8 (9.7)	0.5
Duration of surgery (min)	90 (45–210)	90 (30–230)	0.5
Duration of anesthesia (min)	150 (75–300)	150 (60–350)	0.69

**Table 6:** Perioperative complication rates in dogs in the Vet-ERAS and CONTROL groups. * Significant difference between the two groups (*p* < 0.05).

	Vet-ERAS (n = 59)	CONTROL (n = 82)	*p* Value
Intraoperative complications (n, %):			
▪ Tachycardia	19 (32.2)	51 (62.2)	0.0004 *
▪ Hypotension	38 (64.4)	50 (61)	0.2
▪ Hypothermia	16 (27.1)	47 (57.3)	0.0004 *
Postoperative major complications (n, %):	12 (20.3)	24 (29.3)	0.32
▪ SIRS	8 (15.7)	12 (14.6)	1
▪ Pulmonary complications	8 (15.7)	4 (4.9)	0.1
▪ Acute kidney injury	3 (5)	4 (4.9)	1
▪ Neurologic complications	2 (3.4)	3 (3.6)	1
▪ Surgical re-intervention	2 (3.4)	1 (1.2)	0.56
▪ Hypotension requiring vasopressors	6 (10.2)	15 (18.3)	0.23
Postoperative minor complications (n, %):	27 (45.7)	66 (80.5)	0.0001 *
▪ Vomiting	8 (13.6)	12 (20.3)	1
▪ Diarrhea	10 (17)	21 (25.6)	0.3
▪ Major electrolytes imbalance	5 (8.5)	23 (28)	0.005 *
▪ Anemia	14 (23.7)	27 (33)	0.34
▪ Abdominal pain	7 (11.8)	12 (14.6)	0.8
▪ Fever	9 (15.3)	12 (14.6)	1
▪ Anorexia	27 (45.7)	41 (50)	0.71
▪ Surgical site complications	3 (5)	5 (8.5)	1
▪ Hyperlactatemia	7 (11.8)	14 (24)	0.15
▪ Hypoglycemia	6 (10.1)	7 (13.2)	1
▪ Hyperglycemia	21 (39.6)	36 (62)	0.01 *
▪ Hypoalbuminemia	11 (18.6)	14 (51.8)	0.02 *
No complications (n, %)	27 (45.8)	14 (17)	0.0003 *

**Table 7:** Preoperative physical examination, preoperative laboratory findings, and Charlson comorbidity index (CCI) of dogs in the Vet-ERAS group and CONTROL group. HR, heart rate (beats per minute); RR, respiratory rate (breaths per minute); SAP (systolic blood pressure); T° (rectal body temperature). Data regarding are shown as median and range or mean and standard deviation (SD).

	Vet-ERAS (n = 59)	CONTROL (n = 82)	*p* Value
Preoperative physical examination findings			
▪ HR (bpm)	140 (40–215)	138 (80–247)	0.9
▪ RR (bpm)	40 (12–160)	50 (16–150)	0.76
▪ SAP (mmHg)	131 (40–200)	130 (70–240)	0.35
▪ SI	0.99 (0.33–3.5)	0.98 (0.6–2.85)	0.75
▪ T°C	38.4 (34.9–40.6)	38.4 (33.4–40.1)	0.34
Preoperative laboratory findings			
▪ pH	7.40 (7.16–7.53)	7.38 (6.81–7.48)	0.08
▪ PCO_2_ (mmHg)	35.9 ± 8.6	36.4 ± 9.4	0.75
▪ BE (mmol/L)	−4 (−26.7–5)	−4 (−26.7–5)	0.22
▪ HCO^3−^ (mmHg)	−3.7 (−13.2–20.4)	19.7 ± 4.9	0.07
▪ Glucose (mg/dL)	119 (54–275)	114 (5–356)	0.28
▪ Lactate (mmol/L) Na^+^ (mmol/L)	2.5 (0.6–12.8)	2.1 (0.3–16.6)	0.37
▪ Na^+^ (mmol/L)	142 ± 5.9	144 ± 7	0.21
▪ K^+^ (mmol/L)	3.8 (2.8 –5)	3.72 (2.9–5.1)	0.88
▪ Cl^−^ (mmol/L)	112 (92–122)	113 (92–134)	0.28
▪ Ca^+2^ (mmol/L)	1.25 (0.99–1.4)	1.31 (0.79–1.64)	0.3
▪ WBC count (×10^3^/μL)	14.7 (4.6–46.6)	12.9 (3.8–43)	0.22
▪ Platelet count (×10^3^/μL)	234 (49–666)	246 (8–590)	0.64
▪ Hemoglobin (g/dL)	15.4 ± 4	15.4 ± 4.5	0.97
▪ Total proteins (g/dL)	6.5 ± 1.2	6.6 ± 1.4	0.7
▪ Creatinine (mg/dL)	1 (0.4–10)	0.9 (0.4–12.8)	0.97
▪ Albumin (mg/dL)	2.8 ± 0.7	3 ± 0.7	0.25
ASA physical status	4 (2–5)	4 (2–5)	0.8
CCI (1–3 scale)			
▪ 0 (n, %)	35 (59.3)	16 (19.5)	<0.001
▪ 1 (n, %)	22 (37.3	44 (53.6)	0.06
▪ 2 (n, %)	2 (3.4)	10 (12.2)	0.07
▪ 3 (n, %)	0	0	

**Table 8:** Use of locoregional anesthesia techniques, anti-emetic prophylaxis, feeding tube, and time to first oral intake of dogs in the Vet-ERAS group and CONTROL group. Data regarding first oral intake are shown as median and range. * Significant difference between the two groups (*p* < 0.05).

	Vet-ERAS (n = 59)	Control (n = 82)	*p* Value	Odds Ratio
Locoregional anesthesia techniques (n, %)	42 (71)	30 (36)	<0.0001 *	0.23
Anti-emetic prophylaxis (n, %)	57 (97)	10 (12)	<0.0001 *	0.004
Feeding tube placement (n, %)	39 (66)	22 (27)	<0.0001 *	0.17
Time to first oral intake (hours)	12 (3–48)	23 (4–48)	<0.0001 *	

## Data Availability

The data presented in this study are available in this article.

## Supplementary Materials

The following supporting information can be downloaded at: https://www.mdpi.com/article/10.3390/vetsci12040377/s1, Table S1: Vet-ERAS program for dogs undergoing EL; Table S2: Perioperative surgical safety checklist.
