# Supplementary material for: Veterinary Enhanced Recovery After Surgery (Vet-ERAS) Program in Dogs Undergoing Emergency Laparotomy
Source: Vet Sci. 2025 Apr 17;12(4):377. doi: 10.3390/vetsci12040377 (PMC12030949; doi:10.3390/vetsci12040377)
Supplement: Supplementary file 1 [file vetsci-12-00377-s001.zip › vetsci-3533328-supplementary.pdf]

UNIVERSITÀ DI PISA

Table S1: Vet-ERAS protocol for emergency laparotomy in dogs

Universidad Católica de Valencia San Vicente Mártir

- Use of the **surgical safety checklist** throughout the perioperative period

Hypovolemic shock stabilization

Individualized goal-directed fluid resuscitation, with frequent reassessment until resuscitation endpoints are achieved. Reccomendations:

- **First-line:** Isotonic crystalloids — 20 mL/kg bolus over 10 minutes followed by reassessment. Supplemental 10 mL/kg boluses may be given every 10 minutes, with reassessment, up to a total of 90 mL/kg.
- **Second-line:** Hypertonic saline — 3–8 mL/kg, with a flow rate ≤ 1 mL/kg/min.
- **Oxygen supplementation** by mask

Resuscitation endpoints	
**HR (bpm)**	Small-breed dogs: 100-160<br>Large-breed dogs: 60-100
**RR (bpm)**	20 – 40
**SAP (mmHg)**	> 100
**MAP (mmHg)**	> 70
**Shock index**	< 1
**T (°C)**	37.5 – 39.1
**Lactate blood concentration (mmol/L)**	< 2.5

Dogs must **not be anesthetized** until acceptable resuscitation endpoints are achieved — unless otherwise decided by the attending criticalist (e.g., in cases of active hemorrhage).

Blood transfusion if Hct < 20% or Hgb < 5–6 g/dL.

In cases of active hemorrhage, **lower endpoints** for SAP and MAP may be acceptable:

- SAP ≥ 90 mmHg
- MAP ≥ 50 mmHg

Antibiotic and anti-emetic prophylaxis (Maropitant 1 mg/kg IV or SC).

Blood screening:

- **Blood gas analysis** on admission and after fluid resuscitation if lactate > 2.5 mmol/L.
- **Hematology, biochemistry, and coagulation times** at the discretion of the attending criticalist.

One locoregional anesthesia technique should be performed either before or after surgery.

Locoregional anesthesia techniques	Drugs
**Epidural injection**	Morphine 0.1 mg/kg with or without ropivacaine or bupivacaine 0.5% (max 3 mg/kg)
**Inter-fascial plane blocks (QL, TAP, RSP block)**	Ropivacaine or bupivacaine (3 mg/kg)
**Linea alba infiltration**	Ropivacaine or bupivacaine 0.5% (3 mg/kg)
**Intraperitoneal lavage**	Ropivacaine or bupivacaine 0.5% (3 mg/kg)

Esophagostomy or nasogastric feeding tube should be placed in all dogs deemed critically ill or with a history of anorexia.

First mobilization: At 6 hours post-surgery.

Nutritional plan
First meal to be offered 6-12 hours after surgery.
Voluntary nutrition to be preferred, otherwise feeding tube, if placed.
Three meals per day; RER gradually increased over 2-4 days:<br>1. 33% RER<br>2. 66% RER<br>3. 100% RER

Postoperative analgesia, nutrition, and mobilization must be planned according to the **surgical safety checklist**

First mobilisation at 6 hours post surgery

UNIVERSITÀ DI PISA

Table S2: Surgical safety checklist (Vet-ERAS protocol)

Universidad Católica de Valencia San Vicente Mártir

**BEFORE INDUCTION OF ANESTHESIA**<br><br>Checklist compiled:<br>at the moment □<br>afterwards □	**BEFORE THE START OF SURGERY**<br><br>Checklist compiled:<br>at the moment □<br>afterwards □	**BEFORE RECOVERY**<br><br>Checklist compiled:<br>at the moment □<br>afterwards □	**POSTOPERATIVE PERIOD**
**Owner information**<br>**Owner Information:**<br>☐ Procedure explained<br>☐ Possible complications discussed<br>☐ Cost estimate signed<br>☐ Consent form signed	**Forced air heating system checked**?<br>yes □ no □	**Additional procedures?**<br>Central venous catheter □<br>Feeding tube □	**Handover of information?**<br>yes □ no □<br>**Person in charge of postoperative monitoring __________**
**Pre-anaesthetic evaluation**<br>☐ Blood gas analysis<br>☐ Hematology<br>☐ Biochemistry<br>☐ Coagulation times<br>☐ ECG<br>☐ Thoracic radiography	**Sponge count**<br>yes □ no □<br>Number of sponges _____	**Locoregional anaesthesia technique performed?**<br>yes □ no □<br>Epidural □<br>Quadratus Lumborum block □<br>Trasnversus Abdominis plane block □<br>Rectus sheath plane block □<br>Linea alba infiltration □<br>Peritoneal lavage □	**Analgesic plan?**<br>yes □ no □<br>___________<br><br>**Rescue analgesic plan?**<br>yes □ no □<br>___________
**Physical examination on arrival**<br>HR_____ SAP_____<br>RR_____ MAP_____<br>T°_____ Shock index_____	**Antibiotic prophylaxis given within 60 min before surgery?**<br>yes □ no □<br>Time second dose______	**Postoperative NSDAIDs?**<br>☐ Yes ☐ No<br>Contraindications:<br>☐ Renal failure<br>☐ Hepatic failure<br>☐ Coagulopathy<br>☐ Dehydration<br>☐ Hypotension	**Anti-emetic prophylaxis?**<br>yes □ no □<br>Time administration _____
**Physical examination after stabilisation**<br>HR_____ SAP_____<br>RR_____ MAP_____<br>T°_____ Shock index_____	**Surgery and anaesthesia teams confirm:**<br>☐ Procedure<br>☐ Anticipated complications<br>☐ Cost estimate signed<br>☐ Consent form signed	**Wound dressing applied?**<br>yes □ no □	**Elizabethan collar placed?**<br>yes □ no □
**Blood lactate concentration (mmol/L)**<br>On arrival _____<br>After stabilisation _____	**DURING SURGERY**	**Anticipated risks during recovery?**<br>yes □ no □<br>Management_________	**Blood lactate concentration (mmol/L)**<br>After surgery _____
**Blood transfusion?**<br>yes □ no □<br>Blood typing yes □ no □<br>Blood type _____	**Gastrointestinal surgery sonsiderations:**<br>☐ Second drape placed prior to GI tract incision and removed after GI sutures completed<br>☐ Gloves changed after GI tract closure<br>☐ Separate sterile instruments/table used to close abdominal wall		**Fluid therapy plan?**<br>yes □ no □
**Antibiotic prophylaxis?**<br>yes □ no □<br>Time administration _____<br>Time second dose______	**Sponge count**<br>yes □ no □<br>Number of sponges _____		**Nutritional plan?**<br>yes □ no □
**Anti-emetic prophylaxis?**<br>yes □ no □<br>Time administration _____			**Mobilization plan?**<br>yes □ no □
**Anaesthesia-related material checked?**<br>Laryngoscope and ETT □<br>Anesthetic drugs □<br>Emergency drugs □<br>Anesthesia machine □<br>Monitoring equipment □<br>IV access □<br>Preoxygenation □			**Owner informed?**<br>**Fluid therapy plan?**<br>yes □ no □
